# Multi-step prediction for influenza outbreak by an adjusted long short-term memory

**DOI:** 10.1017/S0950268818000705

**Published:** 2018-04-02

**Authors:** J. Zhang, K. Nawata

**Affiliations:** Department of Technology Management for Innovation, Graduate School of Engineering, The University of Tokyo, 7-3-1 Hongo, Bunkyo-Ku, Tokyo 113-8656, Japan

**Keywords:** Influenza-like illness (ILI), long short-term memory (LSTM), multi-step-ahead time-series prediction

## Abstract

Influenza results in approximately 3–5 million annual cases of severe illness and 250 000–500 000 deaths. We urgently need an accurate multi-step-ahead time-series forecasting model to help hospitals to perform dynamical assignments of beds to influenza patients for the annually varied influenza season, and aid pharmaceutical companies to formulate a flexible plan of manufacturing vaccine for the yearly different influenza vaccine. In this study, we utilised four different multi-step prediction algorithms in the long short-term memory (LSTM). The result showed that implementing multiple single-output prediction in a six-layer LSTM structure achieved the best accuracy. The mean absolute percentage errors from two- to 13-step-ahead prediction for the US influenza-like illness rates were all <15%, averagely 12.930%. To the best of our knowledge, it is the first time that LSTM has been applied and refined to perform multi-step-ahead prediction for influenza outbreaks. Hopefully, this modelling methodology can be applied in other countries and therefore help prevent and control influenza worldwide.

## Introduction

Influenza, commonly known as flu, circulates worldwide and places a substantial burden on people's health every year. The flu outbreak resulted in approximately 3–5 million annual cases of severe illness and 250 000–500 000 deaths [1]. In the USA, annual flu outbreak led to an average of 610 660 life-years lost, 3.1 million hospitalised days and 31.4 million outpatient visits. The total economic burden of annual flu outbreak using projected statistical life values amounted to $87.1 billion [2]. Flu is one of the costliest epidemics worldwide.

The flu vaccine is one of the best ways to reduce the risk of getting sick with flu and spreading it to others [3]. During the 2015–2016 flu season, flu vaccine prevented an estimated 5.1 million illnesses, 2.5 million medical visits, 71 000 hospitalisations and 3000 pneumonia and influenza deaths [3]. However, because flu virus undergoes high mutation rates and frequent genetic re-assortment [4–6], manufacturing flu vaccine suffers from a complicated process every year. In Februaries, World Health Organization (WHO) assesses the strains of flu virus that are most likely to be circulating over the following winter. Then, vaccine manufacturers produce flu vaccines in a very limited time [7]. Usually, the first batch of vaccine is unavailable until Septembers [8, 9].

Moreover, hospital beds assignment to flu patients is also a challenging task due to the limited capacity of hospital beds, time dependencies of bed request arrivals and unique treatment requirements of flu patients [10]. Furthermore, flu seasons vary in timing, severity and duration from one season to another [7]. Therefore, flu hospitalisation also varies by sites and time in each season [11, 12], which makes beds assignment to flu patients more difficult for hospitals.

To help hospitals and pharmaceutical companies better prepare for an annual flu outbreak, we need an accurate model to perform multi-step-ahead time-series prediction for flu outbreaks. Multi-step-ahead time-series prediction, or simply ‘multi-step prediction’, is an analytical task of predicting a sequence of values in future by analysing observed values in the past [13]. Nonetheless, not many past papers studied multi-step prediction for flu outbreaks. The possible reason could be that multi-step prediction usually results in poor accuracy due to some insuperable problems, such as error accumulation. One compromising method is that one can aggregate raw data to a larger time unit and then use the single-step prediction to avoid performing multi-step prediction. For instance, if raw data are weekly based, we can aggregate weekly values to monthly values and then perform a single-step prediction for the coming month (that is roughly around 4 weeks). Although the single-step prediction avoids poor accuracy, it will hinder us from understanding the trend and variation during the coming month.

In this study, we leveraged the deep learning model of long short-term memory (LSTM). Our selection of LSTM was based on the theoretical and practical consideration. In theory, the LSTM is a special kind of RNN. Its elaborate structure (multilayers and gated cells) enables LSTM to learn simulate non-linear function, long-term dependencies [14] and refine time-series prediction [15]. In practice, we found that LSTM achieved the best accuracy in all the six models (autoregressive integrated moving average, support vector regression, random forest, gradient boosting, artificial neural network and LSTM) when we performed a single-step prediction for the US flu data (the same data source with those of this study) in one of our previous studies [16].

## Methods

### Source data and metrics

We used the US flu data from the 40th week of 2002 to the 30th week of 2017, collected from the ‘FluView’ Portal of Centre for Disease Control and Prevention (CDC) [17]. To remove any possible variations in populations, we used the influenza-like illness (ILI) rates as the response (*y*) of models.
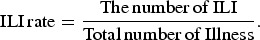


**Fig. 1.**
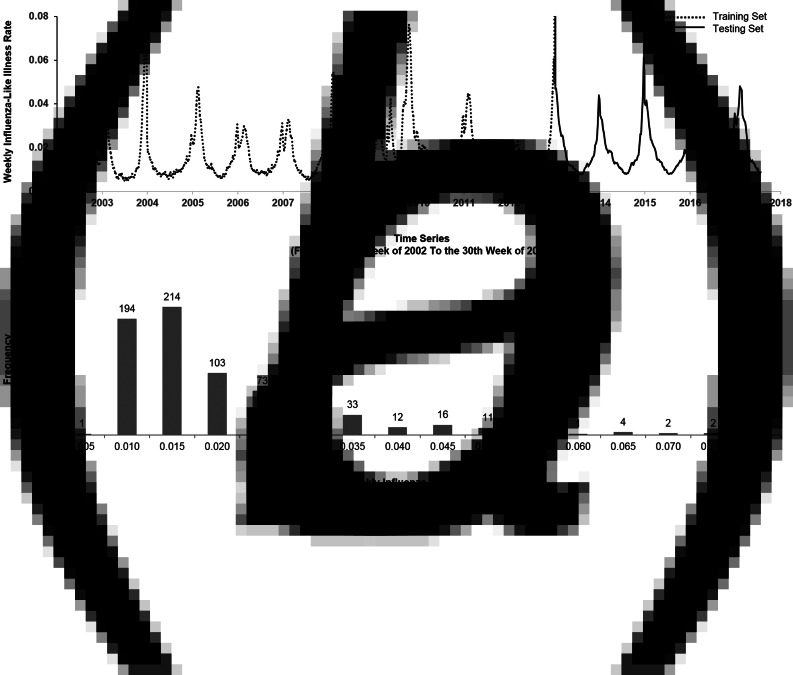
The US flu data from the 40th week of 2002 to the 30th week of 2017. (a) We split the data into the training set and the testing set. The *y*-axis represents the weekly ILI rates, and the *x*-axis represents the time series (from the 40th week of 2002 to the 30th week of 2017). The dashed line is the first 2/3 of the data (from the 40th week of 2002 to the 52nd week of 2012) that were used for training, and the solid line is the last 1/3 of the data (from the first week of 2013 to the 30th week of 2017) that were used for testing. (b) The histogram of the weekly ILI rates of the US flu data. The *y*-axis represents the frequency, and the *x*-axis represents weekly ILI rates. The histogram is right-skewed.

Figure 1(a) illustrates the historical plot of the US flu data. We split the duration into two parts: the first 2/3 (from the 40th week of 2002 to the 44th week of 2012) for training and the last 1/3 (from the 45th week of 2012 to the 30th week of 2017) for testing.

Figure 1(b) describes the histogram of the weekly ILI rates. The histogram is right-skewed. Generally, comparing accuracy of models by mean absolute percentage error (MAPE) mainly reflects the difference of the ‘median’; while comparing by root mean square error primarily reflects the difference of the ‘mean’. The right-skewed histogram and the Kolmogorov–Smirnov test (*P* < 0.05) showed that the US flu data followed a non-normal distribution. Therefore, we expected that the accuracy would reflect the difference of ‘median’ and thereby used MAPE as a key performance indicator (KPI) for comparing models.
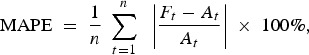
where *A*_*t*_ is the actual value and *F*_*t*_ is the forecast value.

### Feature space and responses

For the feature space, we adopted the time lag of 52 weeks due to the result of one of our previous studies. In the previous study, we used the same US flu data and compared accuarcy of models of the different time lags of 2, 4, 9, 13, 26 and 52 weeks and found that of 52 weeks grew out the best accuracy [16]. Moreover, we calculated the first-order differences as a part of the feature spaces, since some past study found that first-order differences helped improve accuracy of the prediction models for flu data [18]. In brief, for feature spaces, we used (I) the ILI rate of the current week, (II) the ILI rates of the past 52 weeks and (III) the 52 first-order differences. Totally, we have 105 features.

Regarding responses, we were forecasting the two-, three-, four-, five-, six-, seven-, eight-, nine-, 10-, 11-, 12- and 13-step-ahead ILI rates.

### Model

For multi-step prediction, there are mainly two types of methodologies: (I) ‘recursive’ prediction and (II) ‘jumping’ prediction. Generally, the methodology of (I) predicts values step-by-step; the methodology of (II) predicts some-step-ahead values independently. The following (a), (b), (c) and (d) explain four multi-step prediction algorithms [19].

- Multi-stage prediction (MSP)

MSP is a ‘recursive’ prediction. MSP uses one single-output model, which is recursively applied in multiple-step prediction, feeding through the previous output as its new input [20]. In this study, as the first step, we predict *X*_*t*+1_ using the 53 historical values, i.e. *X*_*t*_, *X*_*t*−1_, *X*_*t*−2_, … and *X*_*t*−52_; as the second step, we predict *X*_*t*+2_ based on *X*_*t*+1_ (*X*_*t*+1_ was predicted in the first step), *X*_*t*_, *X*_*t*−1_, *X*_*t*−2_, … and *X*_*t*−51_; as the third step, we predict *X*_*t*+3_ based on *X*_*t*+2_ (*X*_*t*+2_ was predicted in the second step), *X*_*t*+1_ (*X*_*t*+1_ was predicted in the first step), *X*_*t*_, *X*_*t*−1_, *X*_*t*−2_, … and *X*_*t*−50_, etc. Formula 1 describes the prediction process.

*Formula 1. Algorithm of MSP*

prediction_(*t*+1)_ = LSTM_MSP_MODEL#01(observation_(*t*)_, observation_(*t*−1)_, observation_(*t*−2)_, …, observation_(*t*−52))_

prediction_(*t*+2)_ = LSTM_MSP_MODEL#01(prediction_(*t*+1)_, observation_(*t*)_, observation_(*t*−1)_, …, observation_(*t*−51))_

prediction_(*t*+3)_ = LSTM_MSP_MODEL#01(prediction_(*t*+2)_, prediction_(*t*+1)_, observation_(*t*)_, observation_(*t*−1)_, …, observation _(*t*−50))_

…

prediction_(*t*+13)_ = LSTM_MSP_MODEL#01(prediction _(*t*+12)_, prediction_(*t*+11)_, prediction_(*t*+10)_, …, prediction_(*t*+1)_, observation_(*t*)_, observation_(*t*−1)_, …, observation_(*t*−40))_

1. Adjusted multi-stage prediction (AMSP)

AMSP is a refined version of MSP. Comparatively, when AMSP predicting *X*_*t*+*p*_ (*P* ⩾ 2), it uses another model instead of using the same model repeatedly. Such a modification helps suppress error accumulation [21, 22]. Formula 2 illustrates the algorithm of AMSP.

*Formula 2. Algorithm of AMSP*

prediction_(*t*+1)_ = LSTM_AMSP_MODEL#01(observation_(*t*)_, observation_(*t*−1)_, observation_(*t*−2)_, …, observation_(*t*−52))_

prediction_(*t*+2)_ = LSTM_AMSP_MODEL#02(prediction_(*t*+1)_, observation_(*t*)_, observation_(*t*−1)_, …, observation_(*t*−51))_

prediction_(*t*+3)_ = LSTM_MSP_MODEL#03(prediction_(*t*+2)_, prediction_(*t*+1)_, observation_(*t*)_, observation_(*t*−1)_, …, observation_(*t*−50))_

…

prediction_(*t*+13)_ = LSTM_AMSP_MODEL#13(prediction_(*t*+12),_ prediction_(*t*+11)_, prediction_(*t*+10)_, …, prediction_(*t*+1)_, observation_(*t*)_, observation_(*t*−1)_, …, observation_(*t*−40))_

1. Multiple single-output prediction (MSOP)

MSOP is a ‘jumping’ prediction. MSOP directly predicts a *p*-step-ahead (*P* ⩾ 2) value only by historical values: *X*_*t*_, *X*_*t*−1_, *X*_*t*−2_, …, *X*_*t*–*n*_. Formula 3 explains the algorithm of MSOP.

*Formula 3. Algorithm of MSOP*

prediction_(*t*+1)_ = LSTM_MSOP_MODEL#01(observation_(*t*)_, observation _(*t*−1)_, observation_(*t*−2)_, …, observation_(*t*−52))_

prediction_(*t*+2)_ = LSTM_MSOP_MODEL#02(observation_(*t*)_, observation_(*t*−1),_ observation_(*t*−2)_, …, observation_(*t*−52))_

prediction_(*t*+3)_ = LSTM_MSOP_MODEL#03(observation_(*t*)_, observation_(*t*−1)_, observation_(*t*−2)_, …, observation_(*t*−52))_

…

prediction_(*t*+13)_ = LSTM_MSOP_MODEL#13(observation_(*t*)_, observation_(*t*−1)_, observation_(*t*−2)_, …, observation_(*t*−52))_

1. Multiple-output prediction (MOP)

MOP can be regarded as a merged version of MSOP. MOP uses one model to predict many some-step-ahead values all at once. In other fields, MOP was also implemented by multiple support vector regression [20, 23, 24]. Formula 4 outlines the algorithm of MOP.

*Formula 4. Algorithm of MOP*

prediction_(*t*+1)_, prediction_(*t*+2)_, …, prediction_(*t*+13)_ = LSTM_MOP_MODEL#01(observation_(*t*)_, observation_(*t*−1)_, observation_(*t*−2)_, …, observation_(*t*−52))_

### Coding

We used Python and Keras package (Version 2.0.4) [25] based on Tensorflow (Version 1.1.0) [26]. We adopted an ‘early-stopping’ algorithm with a ‘patience’ of 100 epochs (for a total of 1000 epochs) and compared the predicting accuracy of LSTM models of the number of layers: 3–6 and 10.

## Results

### Results of MSP

**Fig. 2.**
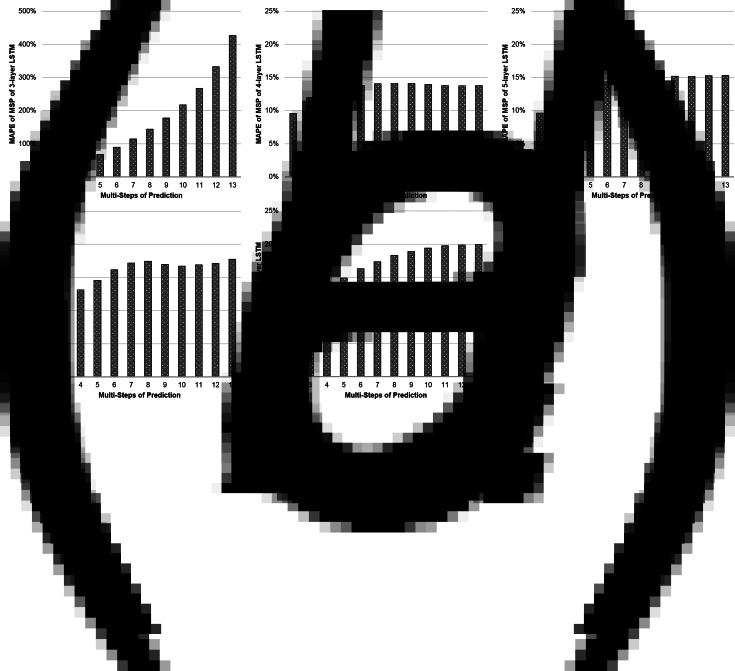
The MAPEs of LSTM with MSP. The *y*-axis represents the MAPE of the predictions and the *x*-axis represents multi-steps of the predictions. The (a–e) illustrate the MAPEs with the MSP algorithm of three-, four-, five-, six-, 10-layer LSTM, respectively.

Table 1(a) illustrates the MAPEs of LSTM with MSP algorithm. The three-layer LSTM with MSP algorithm achieved the predicting MAPEs of 19.757, 32.969, 49.096, 67.866, 89.892, 114.977, 143.999, 177.719, 217.721, 267.335, 332.577 and 426.828%, when forecasting the ILI rates of the coming second to 13th weeks. The MAPE increased by nearly 22 times as the number of predicting steps increased. Comparatively, the MAPEs of the four-layer LSTM with MSP algorithm increased limitedly, from 9.57% to 13.77% with some slight setbacks in 10-, 11- and 12-step prediction. The similar phenomena occurred in the MAPEs of LSTM of five, six and 10 layers with MSP (Fig. 2).

**Table 1.** The MAPEs of LSTM with the multi-step predicting algorithms of MSP, AMSP, MSOP and MOP

(a) The MAPEs of LSTM with the MSP algorithm						
The numbers of multisteps	MAPEs of MSP of three-layer LSTM (%)	MAPEs of MSP of four-layer LSTM (%)	MAPEs of MSP of five-layer LSTM (%)	MAPEs of MSP of six-layer LSTM (%)	MAPEs of MSP of 10-layer LSTM (%)	Average MAPE of MSP of three-, four-, five-, six-, 10-layer LSTM (%)
2	19.757	9.568	9.604	9.018	9.352	11.460
3	32.969	11.960	11.913	11.482	11.778	16.020
4	49.096	13.256	13.223	13.111	13.512	20.439
5	67.866	13.677	13.928	14.552	14.899	24.984
6	89.892	13.794	14.389	16.172	16.272	30.104
7	114.977	14.077	14.899	17.187	17.354	35.699
8	143.999	14.088	15.098	17.439	18.304	41.786
9	177.719	14.100	15.174	16.979	18.894	48.573
10	217.721	13.930	15.202	16.695	19.408	56.591
11	267.335	13.793	15.136	16.894	19.765	66.585
12	332.577	13.763	15.272	17.135	19.880	79.725
13	426.828	13.770	15.320	17.749	19.931	98.720
Average MAPE of MSP of all multi-steps (%)	161.728	13.315	14.096	15.368	16.612	

MAPEs, mean absolute percentage errors; MSP, multi-stage prediction; LSTM, long short-term memory.

**Table:** 

(b) The MAPEs of LSTM with the AMSP algorithm						
The number of multi-steps	MAPEs of AMSP of three-layer LSTM (%)	MAPEs of AMSP of four-layer LSTM (%)	MAPEs of AMSP of five-layer LSTM (%)	MAPEs of AMSP of six-layer LSTM (%)	MAPEs of AMSP of ten-layer LSTM (%)	Average MAPE of AMSP of three-, four-, five-, six-, 10-layer LSTM (%)
2	8.883	9.287	9.453	9.361	8.870	9.171
3	11.326	11.346	11.312	11.377	11.050	11.282
4	12.655	12.906	13.066	12.974	12.378	12.796
5	13.258	13.572	14.078	13.773	13.482	13.633
6	13.897	13.984	14.556	13.245	13.917	13.920
7	14.747	13.676	14.678	14.325	14.283	14.342
8	15.208	14.548	14.110	14.474	15.390	14.746
9	14.631	13.774	15.500	14.406	15.260	14.714
10	14.908	14.616	14.004	13.702	15.213	14.488
11	14.582	15.142	15.307	14.802	17.347	15.436
12	14.718	15.109	15.247	13.616	15.455	14.829
13	14.702	14.569	15.163	14.849	14.292	14.715
Average MAPE of AMSP of all multi-steps (%)	13.626	13.544	13.873	13.409	13.911	

MAPEs, mean absolute percentage errors; AMSP, adjusted multi-stage prediction; LSTM, long short-term memory.

**Table:** 

(c) The MAPEs of LSTM with the MSOP algorithm						
The number of multi-steps	MAPEs of MSOP of three-layer LSTM (%)	MAPEs of MSOP of four-layer LSTM (%)	MAPEs of MSOP of five-layer LSTM (%)	MAPEs of MSOP of six-layer LSTM (%)	MAPEs of MSOP of ten-layer LSTM (%)	Average MAPE of MSOP of three-, four-, five-, six-, 10-layer LSTM (%)
2	8.759	8.842	8.965	9.110	8.878	8.911
3	10.780	10.492	10.162	10.456	10.712	10.520
4	12.035	12.199	12.365	12.114	11.954	12.133
5	12.990	12.776	13.097	12.978	13.137	12.996
6	13.411	13.315	13.504	12.871	13.688	13.358
7	14.075	14.210	14.087	13.780	13.993	14.029
8	14.557	13.718	14.550	14.189	15.098	14.422
9	14.527	13.726	14.325	14.090	14.669	14.267
10	14.395	13.826	14.222	14.564	15.668	14.535
11	14.725	13.972	14.288	13.299	14.497	14.156
12	13.981	13.809	14.585	13.672	14.256	14.061
13	14.415	14.333	13.450	14.036	14.087	14.064
Average MAPE of AMSOP of all multi-steps (%)	13.221	12.935	13.133	12.930	13.386	

MAPEs, mean absolute percentage errors; MSOP, multiple single-output prediction; LSTM, long short-term memory.

**Table:** 

(d) The MAPEs of LSTM with the MOP algorithm						
The number of multi-steps	MAPEs of MOP of three-layer LSTM (%)	MAPEs of MOP of four-layer LSTM (%)	MAPEs of MOP of five-layer LSTM (%)	MAPEs of MOP of six-layer LSTM (%)	MAPEs of MOP of 10-layer of LSTM (%)	Average MAPE of MOP of three-, four-, five-, six-, 10-layer LSTM (%)
2	11.766	10.163	9.879	10.345	11.581	10.747
3	13.043	13.609	11.728	12.451	12.871	12.740
4	18.158	16.142	13.528	15.252	16.663	15.949
5	19.728	17.442	14.985	19.175	15.292	17.324
6	19.253	18.237	14.882	18.512	24.393	19.055
7	21.221	20.625	16.826	18.690	20.154	19.503
8	20.350	20.560	17.933	20.814	20.864	20.104
9	24.043	24.871	20.444	18.006	23.792	22.231
10	22.487	24.197	21.250	21.708	20.632	22.055
11	22.679	19.278	21.047	22.339	27.194	22.507
12	23.095	24.249	22.869	23.707	26.795	24.143
13	24.532	24.571	29.554	25.307	27.979	26.389
Average MAPE of MOP of all multi-steps (%)	20.030	19.495	17.910	18.859	20.684	

MAPEs, mean absolute percentage errors; MOP, multiple-output prediction; LSTM, long short-term memory.

### Results of AMSP, MSOP and MOP

Tables 1(b–d) display the MAPEs of LSTM with AMSP, MSOP and MOP algorithm. In AMSP, the average MAPE increased from 9.171% to 14.715% as the number of predicting steps increased from two to 13, and varied from 13.626% to 13.911% as the number of layers of LSTM increased from three to 10 layers.

The average MAPEs of LSTM of both MSOP and MOP had a slight upward trend as the number of predicting steps increased (from 8.911% to 14.064% in MSOP; from 10.747% to 26.389% in MOP); and varied limitedly as the number of layers of LSTM increased (from 12.935% to 13.386% in MSOP; from 17.9100% to 20.684% in MOP).

In sharp contrast to MSP, the accuracy of AMSP, MSOP and MOP had little improvement when we used more layers of LSTM.

### Comparison of the average MAPE of MSP, AMSP, MSOP and MOP

**Fig. 3.**
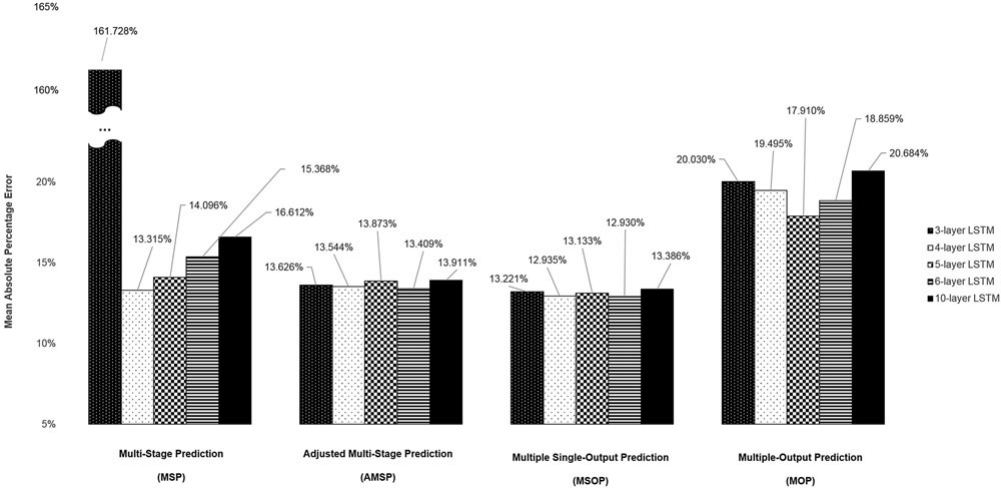
The average MAPEs of LSTM with MSP, AMSP, MSOP and MOP. The *y*-axis represents the MAPE of the predictions and the *x*-axis represents the models of three-, four-, five-, six- and 10-layer LSTM with the multi-step predicting algorithms of MSP, AMSP, MSOP and MOP. Implementing MSOP in the six-layer LSTM achieved the lowest average MAPE of 12.930% in this study.

Figure 3 compares the average MAPEs of LSTM with multi-step predicting algorithms of MSP, AMSP, MSOP and MOP. The different numbers of the layers impacted the predicting accuracy tremendously in MSP (from 13.315% to 161.728%); slightly in MOP (from 17.910% to 20.684%), and barely in AMSP (from 13.626% to 13.911%) and MSOP (from 12.930% to 13.386%). Implementing MSOP in the six-layer LSTM structure achieved the best accuracy in this study. The MAPEs from two-step-ahead to 13-step-ahead prediction for the US ILI rates were all <15%, averagely 12.930%.

## Discussion

### Past studies

We did not find past studies that performed auto-regression in the multi-step prediction for flu outbreaks. Regarding multi-step prediction for studies in other fields, MSP is one of the most popular methodologies probably because many types of models can be used for this purpose, such as linear regression, support vector regression [27], random forest, gradient boosting, artificial neural network [28], etc. However, any such model inevitably introduces errors and tends to suffer from error accumulation problem when the predicted period is long. This is because the bias and variance from previous predictions impact future predictions [20]. These compounding errors change the input distribution for future prediction steps, breaking the train-test independent and identically distributed assumption common in supervised learning [20].

### Comparison of accuracy of MSP, AMSP, MSOP and MOP

When comparing four different multi-step predicting algorithms, we found that the MAPEs of AMSP were less than those of MSP, which demonstrated AMSP suppressed the accumulated errors effectively based on its refined algorithm. Besides, the MAPEs of MSOP are less than those of MOP. As we mentioned in section ‘Methods’, to predict the ILI rates of the coming second to 13th weeks, MOP trained only one model while MSOP trained 13 models. As a result, MSOP can predict with no necessity of sharing neurons in LSTM structure, while MOP has to share neurons in LSTM structure. Consequently, the accuracy of MSOP performed better. Moreover, the average MAPEs of MSOP are slightly less than those of AMSP. The explanation is that MSOP does not accumulate errors at all, while AMSP just adjusted its accumulated errors by training new models. Therefore, MSOP performed best.

### Other features

In our opinion, including other features in multi-step predicting models impacts models’ accuracy positively and negatively. For one thing, when predicting future values, other features could help predict more accurately, especially at turning points, such as an abrupt decrease in temperature. For another thing, before forecasting future ILI rates, we need to forecast other features (e.g. we need weather forecast for temperature and humidity). The error in former prediction could enlarge the error in later prediction. The mechanism is similar to MSP, which accumulates error step by step. In conclusion, whether the accuracy improves or deteriorates might depend on different data in different season from different countries.

In this study, we only performed auto-regression based on two pieces of consideration. First, we regard historical values as a response of all related features, such as temperature, humidity, etc. Therefore, to some extent, taking historical values as feature space includes all related features/factors in models. Besides, how to include temperature or humidity of the whole country in the models is a challenge work. Simply averaging temperature or humidity of all the places (cities and towns) of the USA might bring other problems, such as overlooking in population size, population density, life styles, etc. in different places.

## Conclusion

In this study, we adjusted the LSTM model by the four multi-step prediction algorithms. The result showed that implementing MSOP in a six-layer LSTM structure achieved the best accuracy. The MAPEs from two-step-ahead to 13-step-ahead prediction for the US ILI rates were all <15%, averagely 12.930%. Hopefully, this accurate modelling approach will positively help hospitals, pharmaceutical companies, individuals and governments better prepare for the flu seasons and therefore prevent and control flu outbreaks worldwide.

## References

[1] World Health Organization (WHO) (2017) Influenza (seasonal) act sheet. Available at http://www.who.int/mediacentre/factsheets/fs211/en/ (Accessed 29 October 2017).

[2] Molinari NA, (2007) The annual impact of seasonal influenza in the US: measuring disease burden and costs. Vaccine 25, 5086–5096. 17544181 10.1016/j.vaccine.2007.03.046

[3] Centers for Disease Control and Prevention, National Center for Immunization and Respiratory Diseases (NCIRD) (2016). Estimated influenza illnesses, medical visits, hospitalizations, and deaths averted by vaccination in the United States. Available at https://www.cdc.gov/flu/about/disease/2015-16.htm (Accessed 29 October 2017).

[4] Lubeck MD, Schulman JL and Palese P (1980) Antigenic variants of influenza viruses: marked differences in the frequencies of variants selected with different monoclonal antibodies. Virology 102, 458–462. 6154380 10.1016/0042-6822(80)90114-2

[5] Stech J, (1999) Independence of evolutionary and mutational rates after transmission of avian influenza viruses to swine. Journal of Virology 73, 1878–1884. 9971766 10.1128/jvi.73.3.1878-1884.1999 PMC104428

[6] Suárez P, Valcárcel J and Ortín J (1992) Heterogeneity of the mutation rates of influenza A viruses: isolation of mutator mutants. Journal of Virology 66, 2491–2494. 1548773 10.1128/jvi.66.4.2491-2494.1992 PMC289045

[7] Centers for Disease Control and Prevention, National Center for Immunization and Respiratory Diseases (NCIRD) (2016) Summary of the 2015–2016 influenza season. Available at https://www.cdc.gov/flu/about/season/flu-season-2015-2016.htm (Accessed 29 October 2017).

[8] National Health Service (NHS) (2016) How the flu jab works. Available at https://www.nhs.uk/Conditions/vaccinations/Pages/how-flu-vaccine-works.aspx (Accessed 29 October 2017).

[9] Gerdil C (2003) The annual production cycle for influenza vaccine. Vaccine 21, 1776–1779. 12686093 10.1016/s0264-410x(03)00071-9

[10] Proudlove N, Boaden R and Jorgensen J (2007) Developing bed managers: the why and the how. Journal of Nursing Management 15, 34–42. 17207005 10.1111/j.1365-2934.2006.00632.x

[11] Puig-Barberà J, (2014) First-year results of the global influenza hospital surveillance network: 2012–2013 northern hemisphere influenza season. BMC Public Health 14, 564–575. 24903737 10.1186/1471-2458-14-564 PMC4057821

[12] Centers for Disease Control and Prevention, National Center for Immunization and Respiratory Diseases (NCIRD) (2016) Disease burden of influenza. Available at https://www.cdc.gov/flu/about/disease/burden.htm (Accessed 29 October 2017).

[13] Weigend AS and Gershenfeld NA (1993) Time Series Prediction: Forecasting The Future And Understanding The Past. Santa Fe, New Mexico: Santa Fe Institute.

[14] Hochreiter S and Schmidhuber J (1997) Long short-term memory. Neural Computation 9, 1735–1780. 9377276 10.1162/neco.1997.9.8.1735

[15] Gers FA, Schmidhuber J and Cummins F (2000) Learning to forget: continual prediction with LSTM. Neural Computation 12, 2451–2471. 11032042 10.1162/089976600300015015

[16] Zhang J and Nawata K (2017) A comparative study on predicting influenza outbreaks. Bioscience Trends 11, 533–541. 29070762 10.5582/bst.2017.01257

[17] FluView interactive. Available at https://www.cdc.gov/flu/about/disease/2015-16.htm (Accessed 29 October 2017).

[18] Wu H, (2017) Time series analysis of weekly influenza-like illness rate using a one-year period of factors in random forest regression. Bioscience Trends 11, 292–296. 28484187 10.5582/bst.2017.01035

[19] Brownlee J (2017) Four strategies for multi-step time series forecasting. Available at https://machinelearningmastery.com/multi-step-time-series-forecasting/ (Accessed 29 October 2017).

[20] Cheng H, (2006) Multistep-ahead time series prediction. In Ng WK, Kitsuregawa M, Li J and Chang K (eds). Proceedings of the 10th Pacific-Asia Conference on Advances in Knowledge Discovery and Data Mining. Singapore: Pacific-Asia Conference on Knowledge Discovery and Data Mining, pp. 765–774.

[21] Venkatraman A, Hebert M and Bagnell JA (2015) Improving multi-step prediction of learned time series models. *Proceedings of the Twenty-Ninth Association for the Advancement of Artificial Intelligence Conference on Artificial Intelligence*. Austin: Association for the Advancement of Artificial Intelligence, pp. 3024–3030.

[22] Akhlaghi S and Zhou N (2017) Adaptive multistep prediction based EKF to power system dynamic state estimation. *Power and Energy Conference*. Illinois: Institute of Electrical and Electronics Engineers. doi: 10.1109/PECI.2017.7935748.

[23] Zhang L, (2013) Iterated time series prediction with multiple support vector regression models. Neurocomputing 99, 411–422.

[24] Bao Y, Xiong T and Hu Z (2014) Multi-step-ahead time series prediction using multiple-output support vector regression. Neurocomputing 129, 482–493.

[25] Chollet F (2017) Keras package. Available at https://keras.io/ (Accessed 29 October 2017).

[26] Google Inc. (2017) Available at https://www.tensorflow.org/ (Accessed 29 October 2017).

[27] Muller K, (1997) Predicting time series with support vector machines. In Gerstner W, Germond A, Hasler M and Nicoud JD (eds). International Conference on Artificial Neural Networks. Heidelberg: Artificial Neural Networks, pp. 999–1004.

[28] Narendra KS and Parthasarathy K (1990) Identification and control of dynamical systems using neural networks. Institute of Electrical and Electronics Engineers Transactions on Neural Networks 1, 4–27. 18282820 10.1109/72.80202

